# Predictive and concurrent validity of caregiver reported early childhood development assessments: A prospective cohort study of Tanzanian children

**DOI:** 10.1371/journal.pgph.0006442

**Published:** 2026-08-12

**Authors:** Jonathan Seiden, Dana Charles McCoy, Alfa Muhini, Christopher R. Sudfeld, Mohamed Bakari, Arvin Saleh, Karim P. Manji, Nandita Perumal

**Affiliations:** 1 Department of Leadership, Policy, and Organizations, Peabody College of Education and Human Development, Vanderbilt University, Nashville, Tennessee, United States of America; 2 Harvard Graduate School of Education, Cambridge, Massachusetts, United States of America; 3 Africa Academy for Public Health, Dar es Salaam, United Republic of Tanzania; 4 Department of Global Health and Population, Harvard T.H. Chan School of Public Health, Boston, Massachusetts, United States of America; 5 Department of Internal Medicine, School of Clinical Medicine, Muhimbili University of Health and Allied Sciences, Dar es Salaam, United Republic of Tanzania; 6 Department of Pediatrics and Child Health, Muhimbili University of Health and Allied Sciences, Dar es Salaam, United Republic of Tanzania; 7 Department of Epidemiology and Biostatistics, Arnold School of Public Health, University of South Carolina, Columbia, South Carolina, United States of America; Jawaharlal Nehru Medical College, INDIA

## Abstract

Many directly assessed and caregiver-reported measures of early childhood development (ECD) are used to monitor outcomes and evaluate interventions in low- and middle-income countries (LMICs). Many assessments of ECD for children 0–3 years old have robust psychometric properties, but relatively little is known about their ability to predict later developmental and learning outcomes. To address questions about predictive and concurrent validity, we used longitudinal data on three widely used measures of ECD from 487 children in Tanzania. This study examined the predictive validity of the Caregiver-Reported Early Development Instrument (CREDI) at 12 months of age with the directly assessed International Early Learning and Development Assessment (IDELA) and the caregiver-reported Early Childhood Development Index 2030 (ECDI 2030) administered at 3.5-6 years of age. We also examined the concurrent validity of the IDELA and ECDI2030 administered on the same day. Consistent with prior literature examining the predictive validity of ECD assessments in very young children, we find statistically significant but practically small (~.1 to.2) correlations between age-adjusted CREDI scores measured in infancy and both IDELA and ECDI2030 scores measured at preschool age. Contrary to expectations, CREDI scores were more predictive for children who took IDELA when older and for children whose mothers did not complete primary education. IDELA overall and domain scores had statistically significant medium-sized (~.4 to.5) correlations with ECDI2030 scores that were slightly larger than relations found in prior research. Our results demonstrate limited predictive power from a caregiver-reported assessment administered in infancy, highlighting how variable ECD is for young children. At preschool age, a directly assessed measure of ECD had better reliability than a shorter caregiver-reported measure, but scores on both measures correlated strongly, suggesting that caregiver-reported measures are capturing similar constructs to directly assessed measures of ECD when implemented at the same point in time.

## Introduction

Extensive literature has documented the importance of experiences in early childhood to later life outcomes and the potential for investment in early childhood development (ECD) to translate into long-lasting improvements in children’s later learning, productivity, and wellbeing [1–3]. As efforts to study and improve ECD in Low- and Middle-Income Countries (LMICs) has expanded, the measurement landscape has grown dramatically over the past two decades [4–6]. Assessments vary in terms of the relevant age range, particular domains of development, and modality of administration (e.g., parental or caregiver report of children’s skills/behaviors, direct administration of activities with the child) [4]. As researchers, non-governmental organizations, and governments rely on the results of evaluations using these measures to make decisions about intervention and policy change, it is important to understand how well assessments capture differences in early childhood indicative of durable changes to children’s lives.

Predictive validity is the degree to which a measure at one point at time is able to predict a later outcome, whereas concurrent validity is the degree to which two measures of a similar construct result in similar scores when measured at the same time [7]. Many arguments for ECD investments rest on the assumption that intervention in early childhood translates to benefits later in life [8]. As such, establishing predictive validity is critical. Similarly, a wide range of tools have been used to assess ECD around the world, with practical constraints (e.g., availability of trained data collectors, amount of time with families) often dictating decisions regarding which measures are used. As such, it is also important to assess the concurrent validity of existing measures to ensure that estimates of ECD are not specific to a given measurement approach or modality of assessment (e.g., caregiver report vs. direct assessment).

There is a long history of assessing the predictive and concurrent validity of ECD assessments in high-income countries. In general, this evidence base suggests that: [1] tools used with preschool-aged children have stronger predictive validity for later learning and development than tools used with younger children and [2] there are not consistently better properties for caregiver-reported versus directly assessed tools. For example, in a meta-analysis assessing the predictive validity of preschool screening tools, Sim et al. [9] found strong sensitivity (above 65%) for preschool language measures predicting later delay and Duncan [10] finds that math and reading scores in preschool were the strongest predictors of later school achievement tests with average correlations of.33 and.13 respectively. For tools used with infants and toddlers, the evidence for predictive validity is more mixed. In particular, researchers have raised concerns about these tools under-identifying later cognitive [11] and speech and language delays [12], and providing only modest predictive power of later language skills [13].

Evidence on the predictive and concurrent validity of ECD measures coming from LMIC settings is much more limited [14–16]. The evidence that does exist largely follows trends found in high-income countries. Commonly used assessments for preschool aged (4–6 years old) children have shown strong predictive power for later academic achievement in several LMIC settings, with correlations of around.5 [17,18]. There has been less study on the predictive validity of ECD assessments for children under the age of three, but most researchers find a similar situation to that found in high-income countries: weaker predictive power assessments with younger children than assessments with preschool-aged children. The most significant work is Rubio-Codina and Grantham-McGregor’s [16] comprehensive longitudinal study that examined the predictive power of several commonly used assessments of ECD in Colombia. Scores on ECD assessments administered at age of 19–30 months were modestly correlated (from ~.2 to ~.4) with IQ measured later at six to eight years later, and correlations were stronger for children aged 31–42 months (from ~.3 to ~.5). In contrast, for children 12–18 months old, correlations were very small, and no significant relationships for when children were under the age of 12 months [16]. Other studies have been slightly more encouraging. In a large study in Pakistan, Rasheed et al. [19] found modest (~.3 to ~.4) correlations between scores produced by the directly assessed Bayley Scales of Infant and Toddler Development at two years with Wechsler Preschool and Primary Scale of Intelligence scores at four years. Furthermore, Hamadani et al. [20] found modest correlations (from ~.2 to ~.4) between a parent-reported language measure at 18 months and a measure of IQ administered at age five.

Concurrent validity across modalities of ECD assessment is slightly more studied compared to predictive validity in both high-income contexts [21,22] and in LMICs [14]. For example, in a five-country study Rao et al. [15] found modest (~.3 to ~.5) correlations between short direct assessments and caregiver reports of ECD and Weber et al. [23] found even stronger correlations (~.7) in Senegal.

There is also evidence that both predictive and concurrent validity can vary depending on timing of the assessment and characteristics of the population assessed. Predictive validity has generally been higher when the lag between the first and second assessment is shorter due to less change occurring between the two assessments [16,20]. Concurrent validity between tests may also be stronger for older children and those from more highly educated parents [13,24].

Collectively, this evidence suggests that, in general, ECD assessments used with older toddlers (18 + months) and preschool-age children have modest to strong predictive validity for later measures of learning and development, but that the predictive power for assessments at a younger age are understudied, inconsistent, and often small. Furthermore, although concurrent validity can range greatly depending on the content of the assessment, setting of administration, and child and caregiver characteristics, direct assessments and caregiver reports are almost always at least modestly correlated.

In this study, we build on the existing research by examining the associations of scores on three of the most commonly used ECD assessments internationally: 1) the Caregiver Reported Early Development Instrument (CREDI) [25,26], 2) the directly administered International Development and Early Learning Assessment (IDELA), and 3) the caregiver-reported ECDI2030 [27,28] across time. This allowed us to assess the predictive validity of the CREDI assessed at 12 months along with the convergent validity of the IDELA and ECDI2030 assessed among the same children from 3.5-6 years of age.

## Methods

### Study design and data collection

The data for this study were collected as part of a follow-up study of a randomized, double-blind, placebo-controlled trial that evaluated the efficacy of vitamin D supplementation during pregnancy and lactation among pregnant women living with HIV in Dar es Salaam, Tanzania (ClinicalTrials.gov: NCT02305927) described in detail in Sudfeld et al. [29,30]. The parent trial followed mothers and infants to 12 months of age. Pregnant women were enrolled from July 2015 to April 2018, with the last infant followed up at 12 months of age in October 2019.

The maternal and child cohort was then followed up between February 2022 to March 2023, when children were 3.5 to 6 years of age [31]. Data on child anthropometry and direct ECD assessments, as well as an interviews with the primary caregiver (caregiver-reported ECD assessments, background characteristics, caregiving practices and home learning environments, caregiver mental health, and HIV variables) were collected.

### Ethics statement

The original RCT was approved by the Harvard T.H. Chan School of Public Health Institutional Review Board (IRB13–0231), the Tanzanian National Health Research Ethics Sub-Committee (NIMR/HQ/R.8a/Vol.IX/1658), the Muhimbili University of Health and Allied Sciences Institutional Review Board (2016-05-25/AEC/Vol.X/01), and the Tanzania Food and Drugs Authority (TFDA13/CTR/0005/3). The observational follow-up study was approved by the Tanzanian National Institute of Medical Research (NIMR/HQ/R.8a/Vol. IX/3840), the Muhimbili University of Health and Allied Sciences (DA.282/298/01.C/), and the Harvard TH Chan School of Public Health (IRB21–0994). In both the parent trial and follow-up observational study, mothers or primary caregivers provided written informed consent for participation as described in Sudfeld et al. [29] and Perumal et al. [31].

### Analytical sample

The current methodolodigcal study focuses on 487 mother-child dyads who had complete data on developmental assessments during both the original parent trial and the cohort follow-up. The long recruitment timeframe for the initial intervention study, taking place over three years, combined with follow-up assessments occurring within a single year meant that the age of children at follow-up varied substantially.

### Assessments of ECD

We implemented three assessments of ECD that are commonly used in LMICs. At the Timepoint 1 (T1), when children were on average 12.6 months old (SD = 0.6), we administered items from the Caregiver Reported Early Development Instruments (CREDI) in an interview with the mother of the child. The CREDI is an open-source caregiver-reported assessment that consists of questions about developmental milestones for children aged 0–3 years [26,32]. An earlier version of the CREDI tool was used at T1 in this study, with mothers responding to a fixed form of 41 questions covering Motor, Cognitive, and Language domains. To calculate CREDI scores, we use the CREDI multi-dimensional factor analysis approach to generate both overall and domain scores for children’s Motor, Language, and Cognitive development [33,34].

Data for Timepoint 2 (T2) were collected between 3.5 and 6 years after T1, when children were on average 59.4 months old (SD = 8.9). At T2, we assessed children’s early learning and developmental outcomes with both a directly assessed and caregiver-reported measure. Doing so allowed us to assess the predictive power of CREDI across time, as well as to explore the concurrent relationships between a directly-assessed and caregiver-reported measure during T2. We used the directly assessed International Development and Early Learning Assessment (IDELA) and the caregiver-reported Early Childhood Development Index 2030 (ECDI2030). The IDELA measures school readiness skills within the domains of Early Literacy, Early Numeracy, Motor, Social-Emotional, and Executive Functioning [28,35]. As a direct assessment, a trained enumerator administers 22 standardized activities over the course of about 25–35 minutes to elicit the competencies of the child. The ECDI2030 was designed as a population-level caregiver reported measure of ECD that is the official monitoring instrument of the Sustainable Development Goal 4.2.1 [27,36,37]. It contains a fixed form of 20 questions that can be used to represent children’s overall ECD, and is completed by the child’s primary caregiver (e.g., mother) [38].

### Statistical analysis

Predictive and concurrent validity of ECD assessments are fundamentally questions about correlations of scores across time and assessments. Below we describe how we calculated the ECD assessment scores and the ways in which we measured these correlations to assess the predictive and current validity of these ECD assessments.

### Score creation

Scores on the CREDI, IDELA, and ECDI2030 were calculated in accordance with guidance from the tool creators [34,38,39] to generate continuous factor scores (CREDI), the average percent correct (IDELA), and a sum score (ECDI2030). As with most developmental assessments, these scores are strongly associated with age. To control for the effect of age and instead focus on the relative level of development compared to same-age children, we created internally age-standardized scores. To do so, we fit a regression of predicting the continuous developmental assessment score with a linear term for child’s age. We then extracted the residuals and standardized them into Z-scores such that a score of 0 represents a child scoring at the mean for their age and +/-1 represents a child scoring 1 SD above/below the mean for their age.

Because CREDI scores are generated using item-factor analysis, we were also able to estimate the standard error of measurement (SEM) for each observation. When conducing regression analyses, we follow CREDI guidance to weight all models by the inverse of the squared SEM ($\frac{\text{1}}{SEM^{\text{2}}}$) in order to increase the precision of our results [34]. All statistical analyses were conducted in Stata and R [40,41]. We were unable to evaluate the socio-emotional domain as the version of the CREDI used in this study did not include any specific questions on the socioemotional domain.

### Predictive validity of CREDI

The first step of our analysis was to examine the correlations between CREDI scores at ~12 months of age with IDELA and ECDI2030 scores at 3.5 and 6 years of age. We also assessed whether correlations between scores on similar domains (e.g., Language domain on CREDI and Early Literacy domain of IDELA) were larger than overall correlations.

Predictive validity may also be inflated by other sources of variance beyond the intended construct of ECD. To address this possible confounding, we also examined whether CREDI remains a significant predictor of later ECD outcomes after controlling for child, caregiver, and household characteristics, as shown in equation 1 below. We included demographic controls for the age and sex of the child (as measured at T1), socioeconomic controls for household wealth, education level of the mother, and availability of learning materials in the household, and variables measuring exposure to early stimulation, positive and negative discipline, and neglect. For each ECD Outcome (IDELA or ECDI2030) at T2 of child i, we tested whether the inclusion of additional covariate vector $\beta_{X}$ attenuated the predictive power of CREDI by examining changes in $\beta_{\text{1}}$.


$$\begin{array}{c} {ECD\;Outcome}_{i}=\beta_{\text{0}}+\beta_{\text{1}}CREDI_{i}+\beta_{X}\overset{―}{X_{i}}+\epsilon_{i} \end{array}$$ (1)

### Differences in predictive validity of CREDI

Given previous research on the differential predictive validity of assessments, we used equation 2 to explore whether the predictive power of CREDI differed by three moderators: the age or gender of the child and the educational level of the mother. For each moderator A, we fit:


$$\begin{array}{c} {ECD\;Outcome}_{i}=\beta_{\text{0}}+\beta_{\text{1}}CREDI_{i}+\beta_{\text{2}}A_{i}+\beta_{\text{3}}CREDI_{i}\times A_{i}+\beta_{X}\overset{―}{X_{i}}+\epsilon_{i} \end{array}$$ (2)

In equation 2, $\beta_{\text{3}}$ represents the differential predictive power of CREDI depending on values of A. Regardless of sign, if $\beta_{\text{3}}$ is significant, we have evidence that CREDI scores are differentially predictive of future outcome scores depending on *A*.

### Concurrent validity of IDELA & ECDI2030 at T2

To assess the level of concurrent validity between IDELA and ECDI2030 scores administered at T2, we once again explored pairwise correlations. Additionally, to expand on this analysis, we fit a regression model controlling for the same covariates used in equation 1. In this model, we fit IDELA Overall or Domain-level score j for child i and examined $\beta_{\text{1}}$ to understand the degree to which the association between ECDI2030 scores with IDELA was attenuated after controlling for the vector of covariates $\beta_{X}$. We also examined the internal consistency of both IDELA and ECDI2030 scores by calculating the Cronbach’s alpha coefficient overall and also by age.


$$\begin{array}{c} {IDELA}_{ji}=\beta_{\text{0}}+\beta_{\text{1}}{ECDI\text{2030}}_{i}+\beta_{X}\overset{―}{X_{i}}+\epsilon_{ji} \end{array}$$ (3)

## Results

The descriptive statistics of the sample of 487 mother-child dyads is described in Table 1. While our sample is a non-random sub-sample of the larger study, the mothers included closely resemble the larger sample from the dataset used in Saleh [42]. Compared to the age at T1, where all children were within a month of exactly 12 months old, there was much more variability in the age of the child at T2, ranging from 3.5 to 6.5 years of age, with an average lag of nearly four years between T1 and T2 assessment timepoints.

**Table 1:** Descriptive statistics of sample.

Variable	N	Mean/Proportion	SD
Child’s age (months) at T1 (CREDI)	487	12.6	0.58
Child’s age (months) at T2 (IDELA & ECDI2030)	487	59.4	8.89
Child is female	487	49.9%	
Maternal education: None	486	10.1%	
Maternal education: Primary	486	60.5%	
Maternal education: Secondary	486	23.5%	
Maternal education: Advanced	486	6.0%	
Child is HIV positive at T2	487	2.7%	
Family Care Index (number of stimulating activities out of 6 engaged in with child in last week) at T2	487	4.54	1.86
Positive discipline activities in last week (out of 3) at T2	487	1.12	1.14
Types of physical discipline used in last week (out of 3) at T2	487	1.10	1.17
Child left alone or with another child for more than an hour in last week at T2	487	13.1%	

### Predictive validity of CREDI

**Fig 1:**
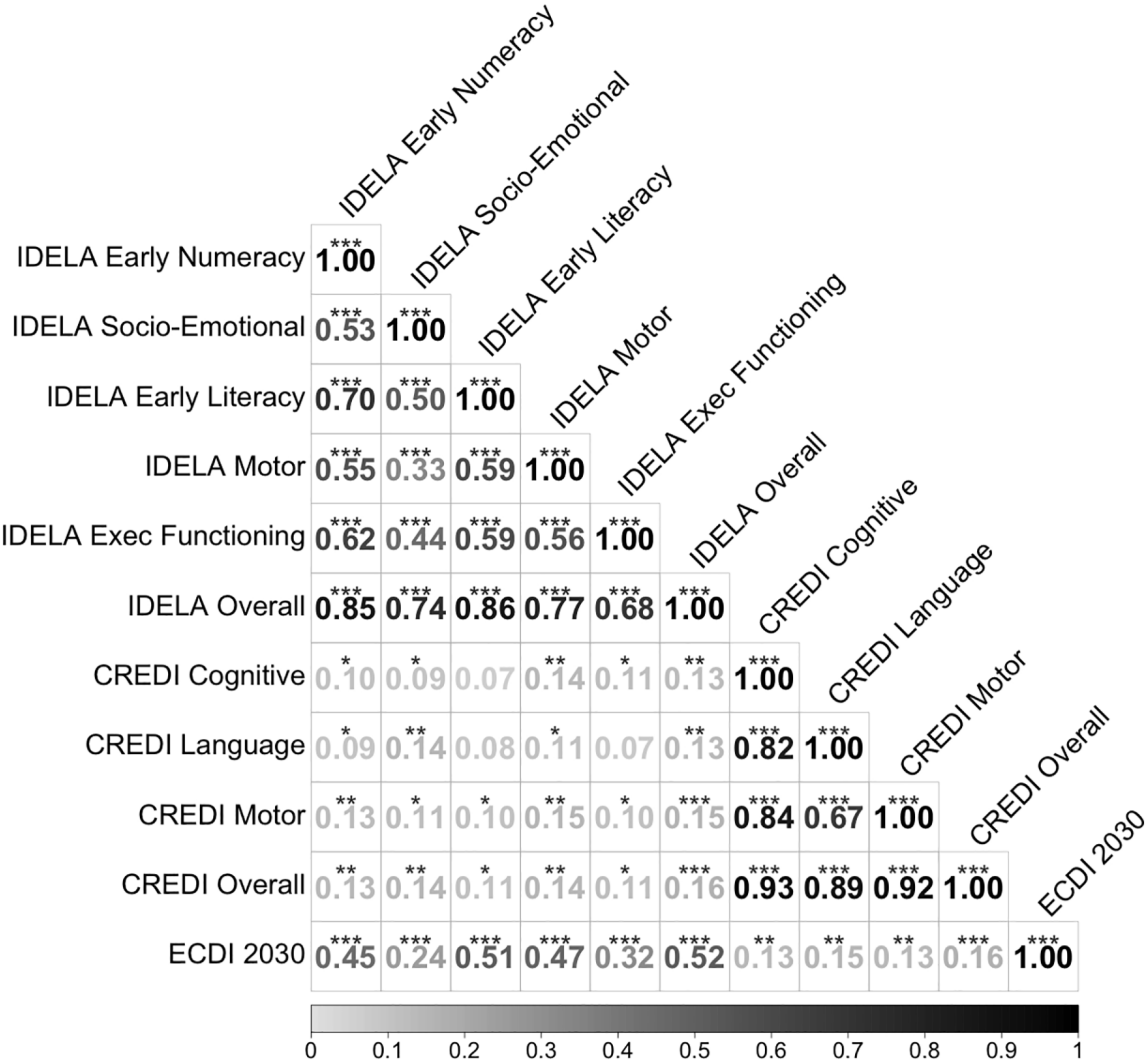
Correlation of CREDI Scores at ~12 months with IDELA and ECDI2030 Age-Normalized Scores at 3.5-6 years (n = 487).

The correlations between the overall and domain scores of CREDI, overall and domain scores of IDELA, and ECDI2030 scores are shown in the correlation matrix in Fig 1. CREDI scores showed a significant, positive correlation with both IDELA and ECDI2030 scores years later, but the absolute magnitude of the relation was small for both assessments at T2 (<0.2). We found little evidence that similar domains correlated more strongly over time. Across all domains of CREDI, the associations with IDELA were small and ranged from about.08 to.17. We find similar results for correlations between ECDI2030 and each of the CREDI domain scores, with no correlations above 0.2.

After examining the bivariate correlations, we fit a regression model to test whether correlations between CREDI at T1 and ECDI2030 and IDELA T2 could be explained by confounding variables. The full results of these regression models are presented in the Supplemental Materials (Table A in S1 Appendix) and estimates of $\beta_{\text{1}}\;$are presented below in Table 2.

**Table 2:** Coefficients of CREDI Overall Score for regression models with and without controls.

*Panel A: Models Predicting IDELA Overall Score*				
	(A1)	(A2)	(A3)	(A4)
	Univariate model	Child controls	Child + SES Controls	Child + SES + Caregiving Controls
CREDI Overall	0.18***	0.18***	0.14**	0.14**
	(0.047)	(0.047)	(0.047)	(0.047)
				
Observations	487	487	486	486
R2	0.03	0.05	0.10	0.11
***Panel B: Models Predicting ECDI2030***				
	**(B1)**	**(B2)**	**(B3)**	**(B4)**
	**No Controls**	**Child controls**	**Child + SES Controls**	**Child + SES + Caregiving Controls**
CREDI Overall	0.16^***^	0.15^***^	0.12^**^	0.11^**^
	(0.042)	(0.041)	(0.041)	(0.041)
				
Observations	487	487	486	486
*R* ^2^	0.03	0.07	0.12	0.14

*Note: Standard errors in parentheses;* ^*^ *p < 0.05,* ^**^ *p < 0.01,* ^***^ *p < 0.001*

As shown in Panel 1 of Table 2, adding child, socioeconomic status, and caregiving controls slightly attenuated the esimated association between CREDI at T1 and IDELA at T2, moving from an estimate of 0.18 in Model A1 with no controls to 0.14 in Model A4 including child, SES, and caregiving controls. As similar pattern is shown in Panel 2, with the association between CREDI and ECDI2030 shifting from 0.16 in Model B1 to 0.11 in Model B4. However, while slightly attenuated, CREDI remained a statistically significant predictor of IDELA at preschool age in all models. This demonstrates that while the association between ECD scores over time is fairly small, it cannot be fully explained by other other environmental and demographic characteristics.

### Differences in predictive validity of CREDI

Next, we explored whether the association between CREDI and later ECD outcomes varied by the sex of the child, the age of the child at T2, or the educational level of the mother. These models continued to control for the same covariates as in Model A4 and B4 above. Significant interactions between CREDI and these variables, regardless of sign, indicated that CREDI was differentially predictive of outcomes. Table 3 presents the main effects and interaction terms for each of these variables for IDELA and ECDI2030. We found no differences in the association between children’s CREDI scores at T1 and ECD outcomes at T2 by child sex. However, we identified statistically significant differences in predictive validity according to the age of the child at T2 and the level of education of the mother.

**Table 3:** Regressions examining the differential predictive power of CREDI at age ~ 12 months on IDELA and ECDI2030 at 3.5-6.5 years.

*Panel 1: Regression models of CREDI Overall predicting IDELA Overall*			
	(C1)	(C2)	(C3)
	Child’s Sex	Age at T2	Maternal Education
CREDI Overall	0.20^**^	0.15^**^	0.56^***^
	(0.066)	(0.047)	(0.17)
			
Age at T2 (months centered on mean)	-0.00004	-0.00090	0.000190
	(0.0053)	(0.0056)	(0.0052)
			
Mother completed primary education	0.25	0.24	0.20
	(0.15)	(0.15)	(0.15)
			
Child is female	0.30^**^	0.29^**^	0.31^***^
	(0.092)	(0.092)	(0.092)
			
CREDI Overall X Child is female	-0.093		
	(0.092)		
			
CREDI Overall X Age at T2		0.012^*^	
		(0.0050)	
			
CREDI Overall X Mother primary education			-0.44^*^
			(0.16)
			
Observations	487	487	487
*R* ^2^	0.104	0.111	0.114
***Panel 2: Regression models of CREDI Overall predicting ECDI2030***			
	**(D1)**	**(D2)**	**(D3)**
	**Child’s Sex**	**Age at T2**	**Maternal Education**
CREDI Overall	0.11	0.12^**^	0.50^***^
	(0.057)	(0.041)	(0.15)
			
Age at T2 (months centered on mean)	-0.0027	-0.0029	-0.0025
	(0.0046)	(0.0046)	(0.0046)
			
Mother has completed primary education	0.13	0.13	0.10
	(0.13)	(0.13)	(0.13)
			
Child is female	0.39^***^	0.39^***^	0.40^***^
	(0.080)	(0.080)	(0.080)
			
CREDI Overall X Child is female	0.012		
	(0.081)		
***Panel 2: Regression models of CREDI Overall predicting ECDI2030***			
	**(D1)**	**(D2)**	**(D3)**
	**Child’s Sex**	**Age at T2**	**Maternal Education**
CREDI Overall X Age at T2		0.0025	
		(0.0044)	
			
CREDI Overall X Mother primary education			-0.41^**^
			(0.15)
			
Observations	487	487	487
*R* ^2^	0.13	0.13	0.14

*Note: Standard errors in parentheses;* ^*^ *p < 0.05,* ^**^ *p < 0.01,* ^***^ *p < 0.001*

**Fig 2:**
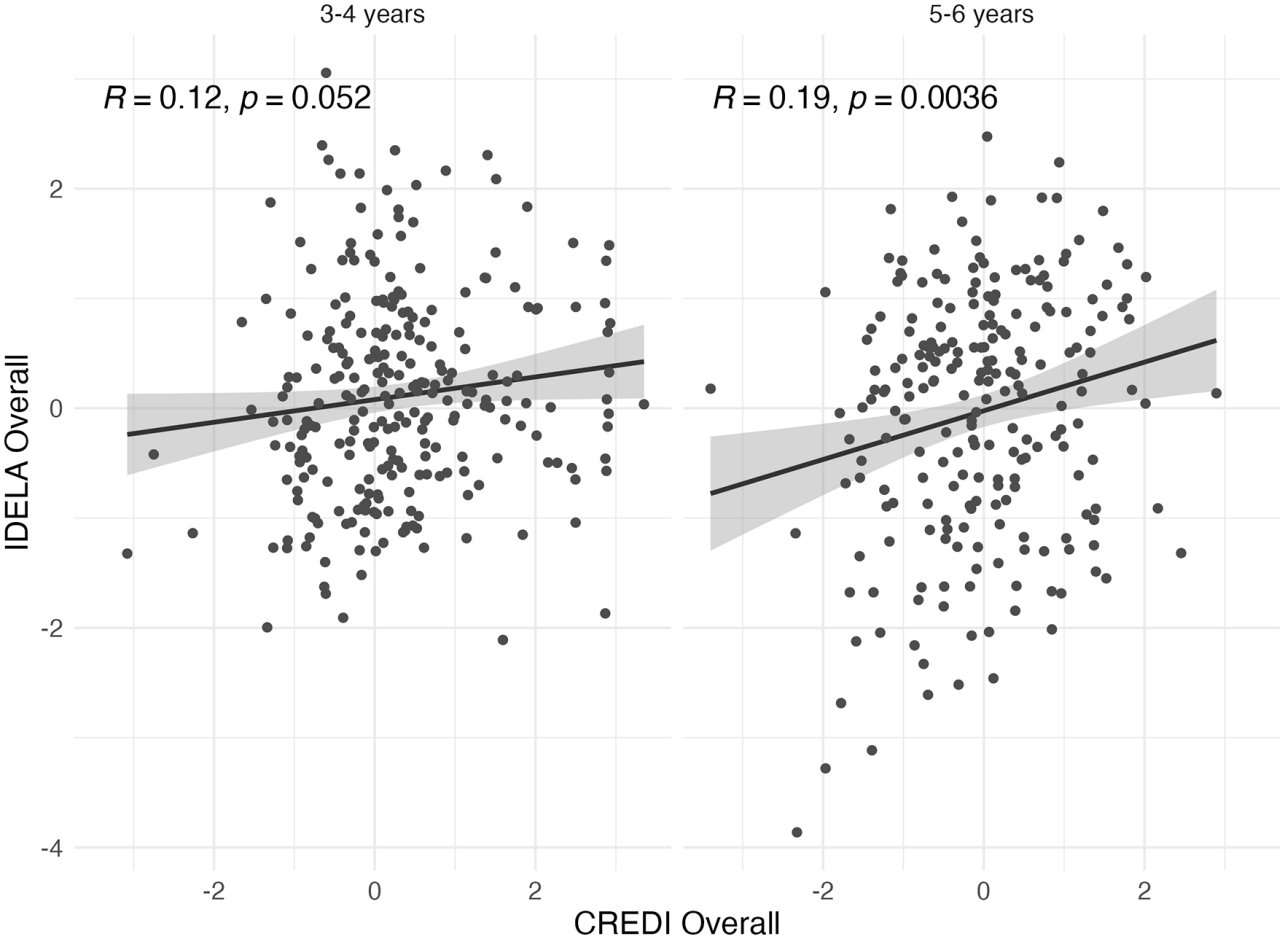
Scatter plot of CREDI and IDELA scores by child’s age at T2.

Specifically, CREDI scores at T1 were more predictive of ECD scores at T2 when administered to older children (i.e., when the amount of time between T1 and T2 was greater) as shown in Fig 2. For IDELA, this difference was statistically significant, as shown in Model C2 of Table 3, where a one-month difference in child age at T2 was associated with a (β=0.01, p=0.023) difference in the predictive power of the CREDI on later IDELA scores. While the corresponding coefficient for ECDI2030 was positive, it was very small and not statistically significant (β=0.00, N.S.).

**Fig 3:**
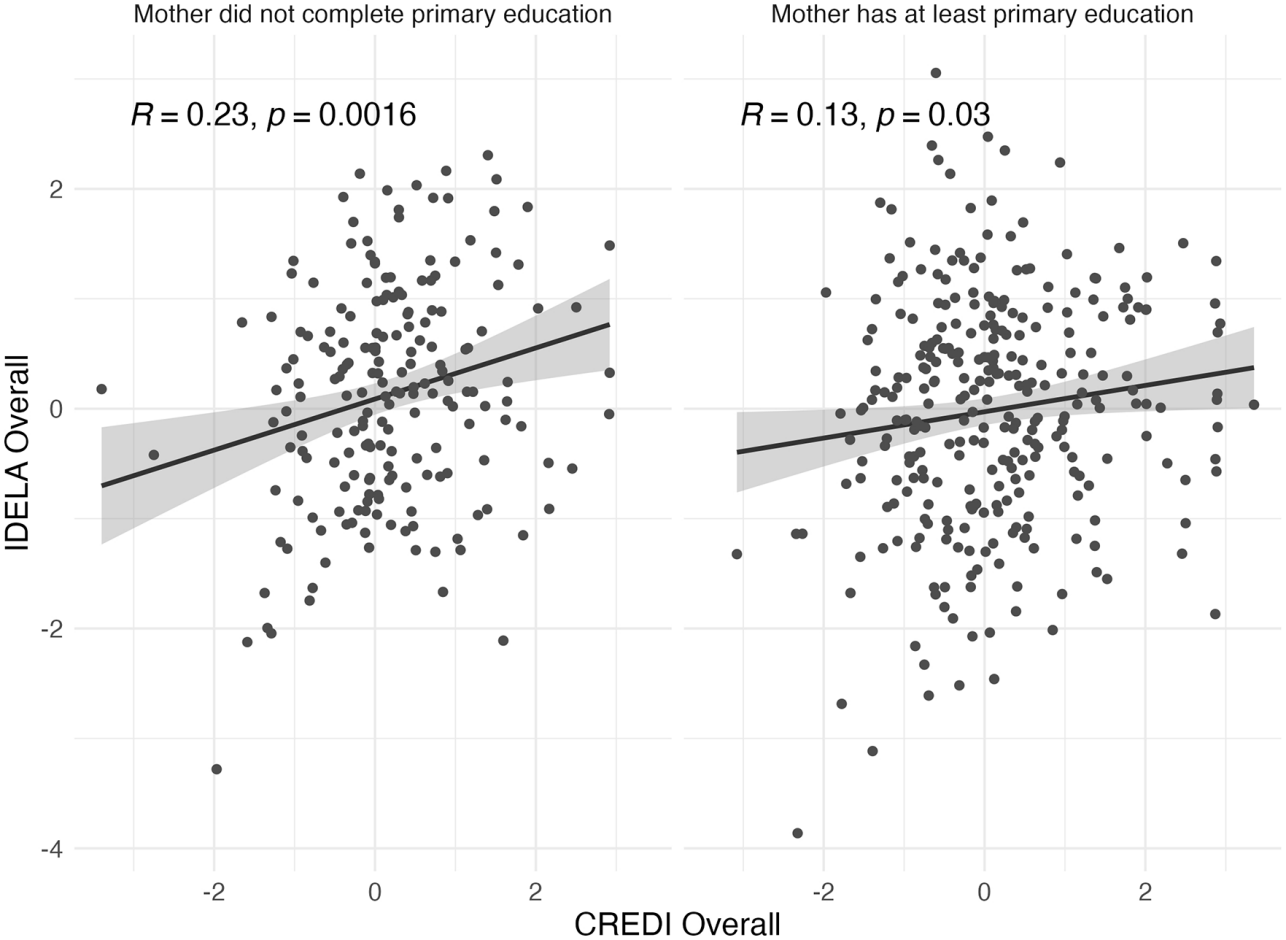
Scatter plot of CREDI and IDELA scores by maternal education level.

We also found a significant interaction between the mother’s education level and the predictive power of CREDI scores on both the ECDI2030 and IDELA scores, as shown in Models C3 and D3 in Table 3. Children whose mother had not competed primary education had a large association with IDELA (β=0.56, p=0.001) and ECDI2030 (β=0.50, p=0.001). The magnitude of the relation was significantly smaller for children whose mother *had* completed primary education, both for IDELA (β=−0.44, p=0.01) and for ECDI2030 (β=−0.41, p=0.007). This finding is visualized in Fig 3, where the relationship between CREDI and IDELA is clearly stronger among children with mothers who had not completed primary education.

### Concurrent validity of IDELA & ECDI2030 at T2

The IDELA and ECDI2030 were both administered on the same occasion to the same children. As expected, for a longer assessment with many more items, IDELA had much higher internal consistency (as measured by Cronbach’s alpha) than the ECDI2030. Across all children, the internal consistency for the IDELA Overall score was 0.93 (excellent) compared with the ECDI2030 at 0.76 (acceptable) [43]. Given the findings from the differential predictive validity, we also calculated the internal consistency by age and maternal education level, as shown in Table 4. IDELA was most reliable for older children (aged five and six), whereas the ECDI2030 was most reliable for four-year-olds. When it comes to maternal education, the IDELA was slightly more reliable for children of mothers without primary education, but ECDI2030 scores were more consistent when reported by mothers who did have at least primary education.

**Table 4:** Reliability of IDELA and ECDI 2030.

*Panel 1: Internal consistency (Cronbach’s alpha) by child’s age at T2*			
Age	N	IDELA	ECDI 2030
3 years old	52	.82	.67
4 years old	209	.90	.75
5 years old	189	.91	.69
6 years old	37	.92	.66
***Panel 2: Internal consistency (Cronbach’s alpha) by maternal education***			
Education	N	IDELA	ECDI 2030
No primary	50	.94	.69
At least primary	437	.93	.76

**Fig 4:**
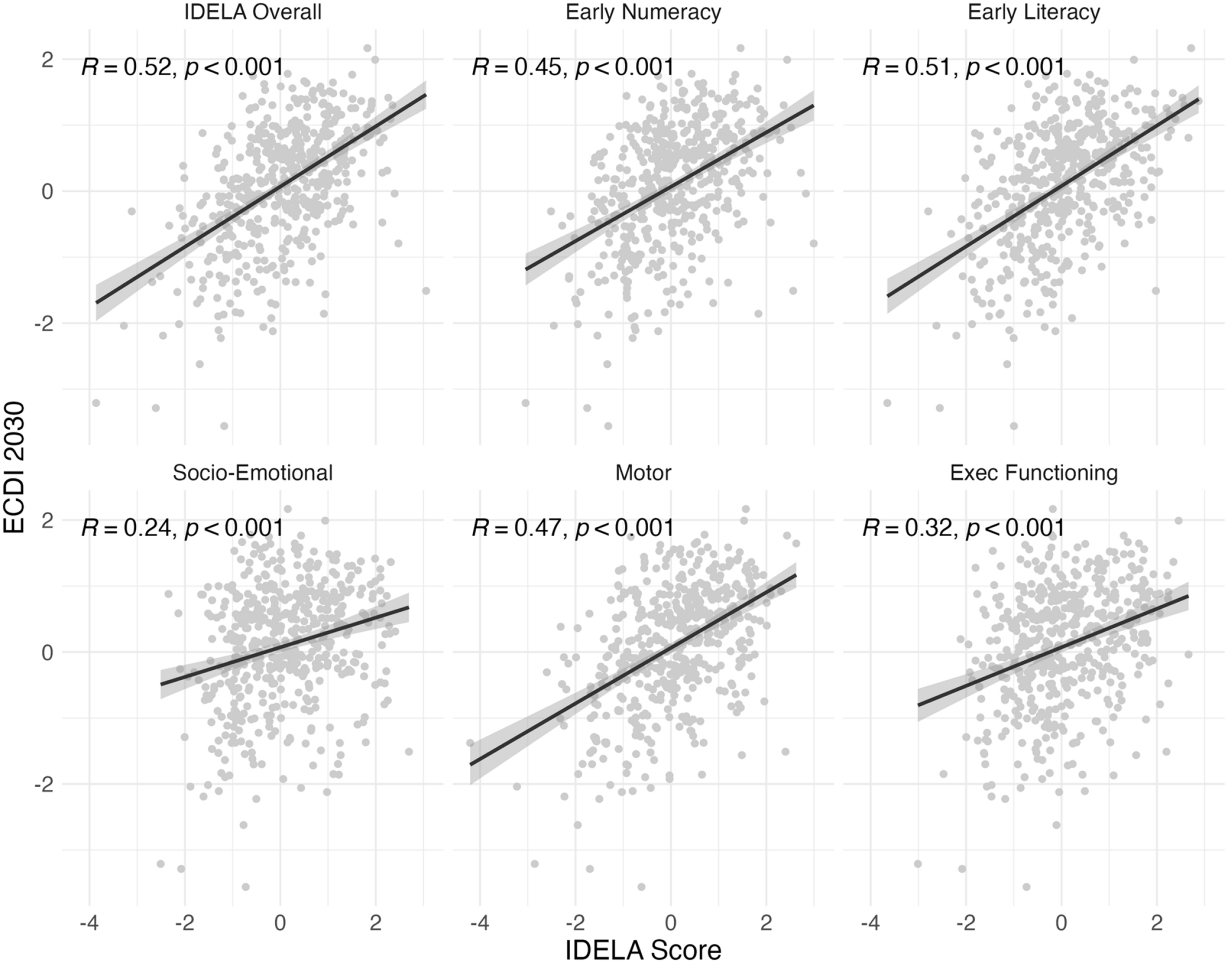
Scatter plot of IDELA domains and ECDI2030.

Despite their differences in reliability, the overall correlations between the IDELA and ECDI2030 indicate generally good concurrent validity for both assessments (R = 0.24 to 0.52). Fig 4 shows the domain-level scatter plots of ECDI2030 and each of the IDELA domains. While all were significantly and positively correlated, the relation was strongest for IDELA’s Early Literacy and Motor domains and smallest for the Executive Functioning and Social Emotional domains, which is in line with expectations given the ECDI2030’s composition of more learning-focused items.

We expanded on this analysis by examining how ECDI2030 and IDELA scores were related in the presence of additional covariates. The full results of this model are reported in the Supplemental Materials (Table A in S2 Appendix) and summarized below in Table 5. Adding control variables to account for confounding demographic variables, socioeconomic status, and caregiving environment only slightly attenuated the relation between ECDI2030 and IDELA. In a model with all controls, the association was only slightly smaller (β=0.54, p<.001) than a model with no additional controls (β=0.57, p<.001).

**Table 5:** Association between ECDI2030 and IDELA Overall Scores across models.

	(1)	(2)	(3)	(4)
	No Controls	Child controls	Child + SES Controls	Child + SES + Caregiving Controls
ECDI2030	0.58^***^	0.57^***^	0.54^***^	0.54^***^
	(0.044)	(0.045)	(0.046)	(0.047)
				
Observations	487	487	486	486
*R* ^2^	0.27	0.27	0.28	0.29

## Discussion

Guided by the premise that investment in early childhood can translate into long-term benefits for children and society, research using measures of ECD make the assumption that changes in these scores are a valid indication of durable changes in children’s lives. As such, empirically testing the predictive power of ECD assessments is critical to understanding the validity and generalizability of findings of research using these measures. Similarly, research that measures ECD through directly assessed or caregiver reported measures also purport to generate evidence on a similar underlying construct. Concurrent validity between measures of ECD in early childhood is required to argue that findings using different modalities are capturing similar results.

### Predictive validity in context

Though limited, previous research on the predictive power of ECD assessments in LMICs at very young ages has indicated generally low or no correlations between scores and learning outcomes measured years later. Our results largely reinforce these prior findings, finding significant but small correlations of about 0.1 to 0.2. These findings are similar to those found by Rubio-Codina & Grantham-McGregor [16], who examined the associations between the “gold standard” (primarily directly assessed) Bayley-III assessment alongside several short tests administered at 6–18 months, with full scale IQ measured at age six to eight years old. Only scores on the Bayley-III were consistently significantly predictive of later IQ, and even these associations were small at about 0.1 to 0.2 [16]. While small, the predictive correlations identified in our study were robust to the inclusion of additional covariates, suggesting that CREDI scores explain unique aspects of variance in child development that persists and is picked up by both a caregiver-reported and directly assessed measure of ECD four years later. Given the relative simplicity and low cost of using the open-source, caregiver-reported CREDI, these findings are encouraging and support continued use of the tool with similar precautions as noted with other assessments [16,19].

Despite the statistical significance of these correlations, the small size of these associations poses important questions regarding the use of ECD assessments with very young children. There are two plausible explanations for these small correlations. A pessimistic view would be to conclude that these measures are highly imprecise and do not capture the aspects of development that are most related to learning and development in later in childhood. A more anodyne explanation is that, rather than reflecting a challenge with measurement, these results reflect early childhood as a period of inherently high variation and large inter-individual differences in developmental trajectories. In the first years of life, children’s development is constantly shifting and adjusting to new environmental inputs, both positive and negative. During this period of heighted neuroplasticity, children have not yet achieved a stable level of individual learning and development [44]. Our findings and previous studies of predictive validity, which have examined a wide array of tools, are more supportive of the second explanation.

### Counterintuitive findings

This research also raises two surprising findings regarding predictive validity. First, we find that that the predictive power of CREDI on both the IDELA and ECDI2030 varied by the education level of the mother. Although we did not have a strong prior to the potential directionality of this interaction, previous research has shown that parental reports by caregivers with lower levels of education may have lower reliability [13], a finding replicated in our study. Despite lower levels of reliability, which in principle should bias correlations towards zero [45], the predictive validity of CREDI scores for these children was higher. We cannot conclusively explain this finding, but a potential mechanism is that mothers with higher education may be more sensitive to their child’s developmental level and intervene more quickly to perceived delays. If higher educated mothers are more responsive the association between children’s earlier and later scores (i.e., the predictive validity of CREDI) will be weaker.

Another surprising finding is that the predictive power of CREDI was *larger* for children who were older at T2 for the directly assessed IDELA, but not for the caregiver-reported ECDI2030. This directly contradicted our expectation that assessments conducted within a relatively short interval would have larger correlations than those that were administered across a longer lag time. This finding may be partially explained by 1) differences in reliability on IDELA and ECDI2030 by age and 2) selection bias that led respondents with later follow-ups at T2 to have more reliable CREDI scores at T1. IDELA scores were most reliable for the oldest children and least reliable for younger children, meaning that T2 scores for IDELA were more reliable for later respondents. The same was not true with ECDI2030—which were most reliable at age 4. We also examined the reliability of CREDI scores by T2 assessment timing and found that those with more reliable CREDI administrations (as measured by the standard error of measurement) were more likely to have a longer follow-up. Because follow-up timing was not randomly assigned, it is possible that more reliable reporters of children’s development were more likely to stay in the study long-term. These two sources of measurement error, their associations with follow-up timing, and the fact that measurement error attenuates correlation coefficients [45], suggest that these counterintuitive findings may be spurious. Despite these possible explanations, these counter-intuitive findings pose important questions that we hope to explore more specifically in more specific studies on differential predictive validity of ECD assessments.

### Concurrent validity in context

The strong correlations between a popular direct assessment of ECD (IDELA) commonly used for impact evaluation and research in LMICs and the ECDI2030, a 20-question caregiver reported measure that is the basis of global monitoring of SDG 4.2.1 also echo earlier findings about concurrent validity. The correlation between the IDELA Overall and ECDI2030 of 0.54 is comparable to the average correlation of 0.48 in Li et al.’s [24] meta-analysis of concordance between parent-reported and directly assessed measures. This suggests that both caregiver-reported and directly assessed measures of ECD are fundamentally assessing a similar construct and that even very brief caregiver reported measures of ECD can generate meaningful data on ECD appropriate for monitoring usage. While direct assessments such as IDELA are typically considered to have better reliability and validity, these correlations demonstrate that, when looking at overall development, short caregiver reported measures that are significantly easier to implement may be sufficient [46]. Despite these overall positive findings of convergent validity, there are substantial differences by domain. Concordance between the measures was strong for early learning but ECDI2030 correlations with Executive Functioning and Social-Emotional development were much weaker.

### Implications for ECD measurement in LMICs and future research

Prior work has identified positive concurrent associations between ECD measures for children under the age of three (e.g., CREDI and others), supporting the notion that scores produced by these measures are accurate indicators of children’s developmental status at a single point of time [33,47]. However, the weak predictive validity of early ECD assessments shown in the current study and others provide important lessons for future researchers studying ECD and other complex developmental constructs [16]. As discussed above, we believe that beyond psychometric challenges, the very nature of development in infancy makes establishing predictive validity of measures difficult. Correlations between measures essentially examine the stability of rankings. Given differing developmental trajectories in the earliest years of life, a child that is relatively undeveloped at one point of time may rapidly catch up either through natural processes or, as our findings suggest might be possible, due to intervention from the caregiver. Weak predictive validity at the individual level may thus reflect genuine variability in the underlying construct. This highlights the importance of repeated measurements when conducting longitudinal research and not relying on a single point of time to make conclusions about an individual or group.

Many studies assume that intervention or policy impacts measured with an ECD assessment in infancy translate to later benefits in life. The predictive validity of an ECD instrument is a starting point to assessing this assumption but does not fully answer the question and weak predictive validity does not necessarily challenge this assumption. To establish whether an ECD assessment detects durable changes in children’s outcomes, future research must examine whether treatment effects on ECD assessments are predictive of treatment effects on later-life outcomes [48]. The finding that effects on ECD assessments, even when measured on assessments with good predictive validity with preschool aged children, can “fade out” over time [49], demonstrates the importance of this question. Future research requires both multiple measures in infancy and a strong intervention effect (or other source of exogenous variation in ECD) in infancy to assess this question.

These findings also have important implications for statistical power calculations when conducting evaluations of interventions. Studies with longitudinal data often use baseline measures to narrow standard errors and improve the precision of estimated effects [50]. Higher correlations between repeated measures improve statistical power more than when they have weak correlations. As such, when conducting power calculation for studies using CREDI or similar measures, assuming a low correlation (about 0.1) will help prevent an underpowered study.

Our concurrent validity findings also demonstrate that short caregiver reported measures like ECDI2030 are appropriate for high-level monitoring use in many situations. However, the limited correlations for some domains show that short unidimensional caregiver reported measures omit some important constructs and are not a substitute for a more comprehensive assessment when differentiation by domain is important. Our findings of meaningful differences in reliability between ECDI2030 and IDELA demonstrate that while direct measures of ECD are time-intensive, they result in scores with increased precision that are more appropriate for use in more detailed analyses and evaluations of interventions.

## Conclusion

Measures of ECD are increasingly used to monitor the well-being of children globally, and to evaluate the effectiveness of interventions in LMICs. Our research on the predictive validity of scores from very young children reinforces earlier research, finding small but statistically significant correlations between scores on a caregiver-reported measure at ~12 months and ECD assessments measured between 3.5-6 years of age. While small, these correlations cannot be explained by other covariates capturing children’s demographics, socioeconomic status, or caregiving environment. We also explored the concurrent validity between two popular measures of ECD for preschool-aged children that capture skills through direct assessment and caregiver report. These findings paint an encouraging picture that, despite differences in reliability, both measures are strongly correlated and capture similar information about the child’s developmental status. Overall, these findings highlight the importance of taking a nuanced and careful approach to measuring ECD and not over-relying on a single measure in infancy for high-stakes decisions.

## Supporting information

S1 Appendix Regression models predicting T2 ECD scores. Table A. Regression models predicting T2 IDELA and ECDI2030 scores with covariates.
(DOCX)

S2 Appendix Concurrent validity regression models. Table A. Regression models predicting IDELA scores with ECDI2030 and covariates.
(DOCX)

S1 File Inclusivity in Global Research Questionnaire. (DOCX)
