# Supplementary material for: Predictive and concurrent validity of caregiver reported early childhood development assessments: A prospective cohort study of Tanzanian children
Source: PLOS Glob Public Health. 2026 Aug 12;6(8):e0006442. doi: 10.1371/journal.pgph.0006442 (PMC13466049; doi:10.1371/journal.pgph.0006442)
Supplement: Supplementary file 1 [file pgph.0006442.s004.pdf]

Review report: PGPH-D-26-01333

Predictive and concurrent validity of caregiver reported early childhood development assessments: a prospective cohort study of Tanzanian children

Journal: PLOS Global Public Health

Authors: Jonathan Seiden, Dana Charles McCoy, Alfa Muhini, Christopher R. Sudfeld, Mohameda Bakari, Arvin Saleh, Karim P. Manji, Nandita Perumal

Summary: the article appears overall adequate and ***acceptable*** for publication. However, here are some suggestions for implementing and improving, if possible, the work done so far.

The study aimed to examine the ability of assessments of children's early psychomotor development to predict developmental and learning outcomes at later ages.

- **Title page**: the title is adequate, concise and contains the essential keywords of the article.
- **Amending the abstract**: it is a short and clear summary of the objectives, methods, results and conclusions and it makes the article interesting to read. Please clarify the abbreviations "*ECD*" and "*LMICs*", specifying that they will appear several times in the article in abbreviated form. Delete all personal pronouns (such as "*we*" on page 2, lines 14, 19, 23; page 3, line 7).
- **Full text**: the research correctly uses the scientific method and was conducted in such a way that the results can be trusted. The paper is comprehensible. In the ***introduction***, please avoid abbreviations, unless they are recurring acronyms — such as "*ECD*" and "*LMICs*"—and always clarify them; if possible, write them out in full. See page 4, lines 7 and 8. Also, remember to eliminate all personal pronouns in the body of the text (see "*we*" on page 6, line 14, and "*us*" on page 6, line 17). Also in the ***methods***, review the personal pronouns "*we*" (page 7, lines 19, 20, 25; page 8, lines 4, 7, 18, 19; page 9, lines 1, 3, 6, 7, 10, 14, 18, 20, 24; page 10, lines 2, 4, 6, 10, 11, 14), "*us*" (page 8, line 5), and "*our*" (page 9, lines 9, 13). Furthermore, the statistical analysis appears well-structured and conducted correctly, even if the reviewer in question is not specialized in this; therefore, if possible, please defer to the relevant person for a double check. In the

results, in addition to eliminating the personal pronouns "*our*" (page 10, line 18) and "*we"* (page 11, lines 7, 9; page 12, line 4; page 13, lines 13, 18, 19; page 14, line 7; page 17, line 6; page 18, line 3), please explain in more detail why the expression "*Error! Reference source not found*" is inserted on pages 12, line 6 and page 18, lines 4 and 5. If this is a typo, please eliminate it and, if necessary, add additional information. In the ***discussion***, the examination of the study's results and the comparison with existing studies on the same topic, cited in the bibliographical references and in the body of the text, is greatly appreciated. However, be careful to always eliminate the personal pronouns "*our*" (page 19, lines 13, 19; page 20, lines 11, 18, 25) and "*we*" (page 20, lines 14, 16, 19; page 21, lines 6, 14). The same should be done in the ***conclusions*** for "*our*" (page 22, line 8) and "*we*" (page 22, line 17).

- **Referencing queries**: work done on the bibliographical references is appreciated.
- **Figures and tables**: all the figures and tables inserted are truly exhaustive of the topics described in the article and are well done and much appreciated, as data and concepts are presented in a clear and appropriate manner.
- **Strenghts and limitations of the study**: in general, outcome measures derived from interviews and/or questionnaires administered to parents/caregivers are generally considered unreliable with respect to the child's actual overall condition. Furthermore, the great variability and unpredictability, as well as the rapid evolution of psychomotor skill development — typical of very young children — must be taken into account when assessing using standardized tests, in order for them to be reliable.

In the future, it might be worth considering repeating the study, implementing it with the *Griffiths III* test, a test for assessing child development similar to the Bayley's scales, as it is directly applied to toddlers and sensitive to even the youngest children's development. Clearly, the study design would need to be rethought and a new sample collected.

The study will be of interest to the readers of PLOS Global Public Health as it contributes to the growing body of evidence about the study of outcome measures in early childhood. If adequately modified with the revisions reported, it will certainly be a valid study acceptable for publication and useful for research in this field.

The reviewer is available for any further clarifications.
