# Supplementary material for: Predictive and concurrent validity of caregiver reported early childhood development assessments: A prospective cohort study of Tanzanian children
Source: PLOS Glob Public Health. 2026 Aug 12;6(8):e0006442. doi: 10.1371/journal.pgph.0006442 (PMC13466049; doi:10.1371/journal.pgph.0006442)
Supplement: Supplementary file 2 [file pgph.0006442.s001.docx]

# S1 Appendix: Regression models predicting T2 ECD scores

# *Table A. Regression models predicting T2 IDELA and ECDI2030 scores with covariates*

*Panel 1: Models Predicting IDELA Overall Scores*

|  | (A1) | (A2) | (A3) | (A4) |
| --- | --- | --- | --- | --- |
|  | No Controls | Child controls | Child + SES Controls | Child + SES + Caregiving Controls |
| CREDI Overall | 0.182^***^ | 0.175^***^ | 0.140^**^ | 0.142^**^ |
|  | (0.0473) | (0.0473) | (0.0469) | (0.0471) |
|  |  |  |  |  |
| Child is female |  | 0.283^**^ | 0.264^**^ | 0.286^**^ |
|  |  | (0.0927) | (0.0916) | (0.0922) |
|  |  |  |  |  |
| Age at IDELA (months centered on mean) |  | -0.00198 | -0.00217 | 0.00000770 |
|  |  | (0.00527) | (0.00522) | (0.00530) |
|  |  |  |  |  |
| SES Wealth Index (SD) |  |  | 0.0823^**^ | 0.0785^**^ |
|  |  |  | (0.0278) | (0.0278) |
|  |  |  |  |  |
| Maternal Education: Primary |  |  | 0.199 | 0.224 |
|  |  |  | (0.156) | (0.156) |
|  |  |  |  |  |
| Maternal Education: Secondary |  |  | 0.358^*^ | 0.356^*^ |
|  |  |  | (0.177) | (0.177) |
|  |  |  |  |  |
| Maternal Education: Advanced |  |  | 0.535^*^ | 0.545^*^ |
|  |  |  | (0.249) | (0.249) |
|  |  |  |  |  |
| Number of types of books & toys child has access to |  |  | 0.0438 | 0.0492 |
|  |  |  | (0.0483) | (0.0488) |
|  |  |  |  |  |
| FCI - Number of Stimulating |  |  |  | -0.0477 |
| Activities (Any Caregiver) |  |  |  | (0.0269) |
|  |  |  |  |  |
| Positive discipline |  |  |  | 0.0678 |
|  |  |  |  | (0.0434) |
|  |  |  |  |  |
| Physical discipline |  |  |  | -0.0455 |
|  |  |  |  | (0.0449) |
|  |  |  |  |  |
| Child left alone or with |  |  |  | 0.125 |
| another child |  |  |  | (0.136) |
|  |  |  |  |  |
| Constant | 0.0331 | -0.108 | -0.436^*^ | -0.301 |
|  | (0.0466) | (0.0654) | (0.182) | (0.222) |
| Observations | 487 | 487 | 486 | 486 |
| *R*^2^ | 0.030 | 0.049 | 0.095 | 0.109 |

Standard errors in parentheses; ^*^ *p* < 0.05, ^**^ *p* < 0.01, ^***^ *p* < 0.001

*Panel 2: Models Predicting ECDI2030*

|  | (B1) | (B2) | (B3) | (B4) |
| --- | --- | --- | --- | --- |
|  | No Controls | Child controls | Child + SES Controls | Child + SES + Caregiving Controls |
| CREDI Overall | 0.155^***^ | 0.146^***^ | 0.116^**^ | 0.112^**^ |
|  | (0.0419) | (0.0414) | (0.0410) | (0.0412) |
|  |  |  |  |  |
| Child is female |  | 0.377^***^ | 0.362^***^ | 0.381^***^ |
|  |  | (0.0811) | (0.0801) | (0.0805) |
|  |  |  |  |  |
| Age at IDELA (months centered on mean) |  | -0.00254 | -0.00191 | -0.00206 |
|  |  | (0.00461) | (0.00457) | (0.00463) |
|  |  |  |  |  |
| SES Wealth Index (SD) |  |  | 0.0901^***^ | 0.0892^***^ |
|  |  |  | (0.0243) | (0.0243) |
|  |  |  |  |  |
| Maternal Education: Primary |  |  | 0.124 | 0.128 |
|  |  |  | (0.136) | (0.136) |
|  |  |  |  |  |
| Maternal Education: Secondary |  |  | 0.164 | 0.132 |
|  |  |  | (0.155) | (0.155) |
|  |  |  |  |  |
| Maternal Education: Advanced |  |  | 0.449^*^ | 0.420 |
|  |  |  | (0.218) | (0.217) |
|  |  |  |  |  |
| Number of types of books & toys child has access to |  |  | 0.0264 | 0.0202 |
|  |  |  | (0.0422) | (0.0426) |
|  |  |  |  |  |
| FCI - Number of Stimulating |  |  |  | 0.0189 |
| Activities (Any Caregiver) |  |  |  | (0.0235) |
|  |  |  |  |  |
| Positive discipline |  |  |  | 0.0748^*^ |
|  |  |  |  | (0.0379) |
|  |  |  |  |  |
| Physical discipline |  |  |  | -0.0252 |
|  |  |  |  | (0.0392) |
|  |  |  |  |  |
| Child left alone or with |  |  |  | 0.238^*^ |
| another child |  |  |  | (0.118) |
|  |  |  |  |  |
| Constant | 0.0754 | -0.112 | -0.303 | -0.463^*^ |
|  | (0.0413) | (0.0572) | (0.159) | (0.193) |
| Observations | 487 | 487 | 486 | 486 |
| *R*^2^ | 0.027 | 0.071 | 0.118 | 0.135 |

*Note: Standard errors in parentheses; ^*^ p < 0.05, ^**^ p < 0.01, ^***^ p < 0.001*

# S2 Appendix: Concurrent validity regression models

# *Table S2.1. Regression models predicting IDELA scores with ECDI2030 and covariates*

|  | (1) | (2) | (3) | (4) |
| --- | --- | --- | --- | --- |
|  | No Controls | Child controls | Child + SES Controls | Child + SES + Caregiving Controls |
| ECDI2030 | 0.582^***^ | 0.573^***^ | 0.541^***^ | 0.540^***^ |
|  | (0.0439) | (0.0451) | (0.0462) | (0.0466) |
|  |  |  |  |  |
| Child is female |  | 0.0693 | 0.0692 | 0.0837 |
|  |  | (0.0829) | (0.0830) | (0.0837) |
|  |  |  |  |  |
| Age at IDELA (months centered on mean) |  | -0.00131 | -0.00172 | 0.000429 |
|  |  | (0.00456) | (0.00459) | (0.00466) |
|  |  |  |  |  |
| SES Wealth Index (SD) |  |  | 0.0404 | 0.0380 |
|  |  |  | (0.0250) | (0.0250) |
|  |  |  |  |  |
| Maternal Education: Primary |  |  | 0.128 | 0.153 |
|  |  |  | (0.139) | (0.139) |
|  |  |  |  |  |
| Maternal Education: Secondary |  |  | 0.265 | 0.280 |
|  |  |  | (0.157) | (0.158) |
|  |  |  |  |  |
| Maternal Education: Advanced |  |  | 0.299 | 0.328 |
|  |  |  | (0.219) | (0.219) |
|  |  |  |  |  |
| Number of types of books & toys child has access to |  |  | 0.0327 | 0.0410 |
|  |  |  | (0.0426) | (0.0431) |
|  |  |  |  |  |
| FCI - Number of Stimulating Activities (Any Caregiver) |  |  |  | -0.0562^*^ |
|  |  |  |  | (0.0238) |
|  |  |  |  |  |
| Positive discipline |  |  |  | 0.0275 |
|  |  |  |  | (0.0386) |
|  |  |  |  |  |
| Physical discipline |  |  |  | -0.0242 |
|  |  |  |  | (0.0391) |
|  |  |  |  |  |
| Child left alone or with another child |  |  |  | 0.0240 |
|  |  |  |  | (0.121) |
|  |  |  |  |  |
| Constant | -0.00649 | -0.0405 | -0.275 | -0.0739 |
|  | (0.0405) | (0.0573) | (0.162) | (0.197) |
| Observations | 487 | 487 | 486 | 486 |
| *R*^2^ | 0.266 | 0.267 | 0.284 | 0.294 |

*Note: Standard errors in parentheses; ^*^ p < 0.05, ^**^ p < 0.01, ^***^ p < 0.001*
