# Supplementary material for: Predictive and concurrent validity of caregiver reported early childhood development assessments: A prospective cohort study of Tanzanian children
Source: PLOS Glob Public Health. 2026 Aug 12;6(8):e0006442. doi: 10.1371/journal.pgph.0006442 (PMC13466049; doi:10.1371/journal.pgph.0006442)
Supplement: Supplementary file 3 [file pgph.0006442.s002.docx]

# S2 Appendix: Concurrent validity regression models

# *Table A. Regression models predicting IDELA scores with ECDI2030 and covariates*

|  | (1) | (2) | (3) | (4) |
| --- | --- | --- | --- | --- |
|  | No Controls | Child controls | Child + SES Controls | Child + SES + Caregiving Controls |
| ECDI2030 | 0.582^***^ | 0.573^***^ | 0.541^***^ | 0.540^***^ |
|  | (0.0439) | (0.0451) | (0.0462) | (0.0466) |
|  |  |  |  |  |
| Child is female |  | 0.0693 | 0.0692 | 0.0837 |
|  |  | (0.0829) | (0.0830) | (0.0837) |
|  |  |  |  |  |
| Age at IDELA (months centered on mean) |  | -0.00131 | -0.00172 | 0.000429 |
|  |  | (0.00456) | (0.00459) | (0.00466) |
|  |  |  |  |  |
| SES Wealth Index (SD) |  |  | 0.0404 | 0.0380 |
|  |  |  | (0.0250) | (0.0250) |
|  |  |  |  |  |
| Maternal Education: Primary |  |  | 0.128 | 0.153 |
|  |  |  | (0.139) | (0.139) |
|  |  |  |  |  |
| Maternal Education: Secondary |  |  | 0.265 | 0.280 |
|  |  |  | (0.157) | (0.158) |
|  |  |  |  |  |
| Maternal Education: Advanced |  |  | 0.299 | 0.328 |
|  |  |  | (0.219) | (0.219) |
|  |  |  |  |  |
| Number of types of books & toys child has access to |  |  | 0.0327 | 0.0410 |
|  |  |  | (0.0426) | (0.0431) |
|  |  |  |  |  |
| FCI - Number of Stimulating Activities (Any Caregiver) |  |  |  | -0.0562^*^ |
|  |  |  |  | (0.0238) |
|  |  |  |  |  |
| Positive discipline |  |  |  | 0.0275 |
|  |  |  |  | (0.0386) |
|  |  |  |  |  |
| Physical discipline |  |  |  | -0.0242 |
|  |  |  |  | (0.0391) |
|  |  |  |  |  |
| Child left alone or with another child |  |  |  | 0.0240 |
|  |  |  |  | (0.121) |
|  |  |  |  |  |
| Constant | -0.00649 | -0.0405 | -0.275 | -0.0739 |
|  | (0.0405) | (0.0573) | (0.162) | (0.197) |
| Observations | 487 | 487 | 486 | 486 |
| *R*^2^ | 0.266 | 0.267 | 0.284 | 0.294 |

*Note: Standard errors in parentheses; ^*^ p < 0.05, ^**^ p < 0.01, ^***^ p < 0.001*
