# Supplementary material for: Predictive and concurrent validity of caregiver reported early childhood development assessments: A prospective cohort study of Tanzanian children
Source: PLOS Glob Public Health. 2026 Aug 12;6(8):e0006442. doi: 10.1371/journal.pgph.0006442 (PMC13466049; doi:10.1371/journal.pgph.0006442)
Supplement: Supplementary file 5 [file pgph.0006442.s006.docx]

For clarity, we have broken up some comments into separate bullets. Comments from the editor/reviewers are in *italics*, while our responses are in **bold** below each bullet.

Editor comments

- *Please ensure that the paper is thoroughly revised in terms of explaining the abbreviations and other typographical errors (as pointed by reviewers) in addition to improvement in discussion.*

**The paper has been checked thoroughly to ensure that all acronyms are spelled out on the first instance. We have also given the paper a thorough proofread to identify and resolve typos (in addition to those identified by the reviewers). We have also updated the Discussion as detailed in our response below.**

- *The discussion is appropriate in terms of the explanation of the results but does not go beyond it, in terms of explaining the wider implications for ECD measures and their use in LMICs. The discussion would be strengthened with the reflection on the measures (including the ones studied) in the presence of other measure; the practical implications of the findings of the caregiver reported childhood measures and also the future directions of research (like exploring the evidence for understanding the pessimistic or optimistic point of views more clearly).*

**This is a fair criticism which has led to the largest change in the manuscript. We have revised the Discussion substantially to first better section it off into more digestible sections and have added a specific section “Implications for ECD measurement in LMICs and future research” beginning on page 24 to address these points specifically.**

- *In conclusion you raise valid points on the variability of measures and how young children's performance is not deterministic but this point has not been well explained in the discussion. I think it warrants a discussion so that the readers can clearly understand what you mean by 'ongoing intervention and support'*

**Another good point. We have moved some of this language to the Discussion section under “Implications for ECD measurement in LMICs and future research” and expanded our explanation of this.**

- *1. Please include a complete copy of PLOS’ questionnaire on inclusivity in global research in your revised manuscript.*

**We have responded to all questions in the “Human Subject Research” part of this questionnaire and uploaded it.**

- *2. Please ensure that your Ethics Statement is available in its entirety at the beginning of your Methods section, under a subheading 'Ethics Statement'.*

**We have consolidated the ethical approvals that the study received in the “Ethics statement” section under Methods.**

- *3. Please amend your detailed Financial Disclosure statement. This is published with the article. It must therefore be completed in full sentences and contain the exact wording you wish to be published.
  a. Please clarify all sources of funding (financial or material support) for your study. List the grants (with grant number) or organizations (with url) that supported your study, including funding received from your institution. 
  b. State the initials, alongside each funding source, of each author to receive each grant.
  c. State what role the funders took in the study. If the funders had no role in your study, please state: “The funders had no role in study design, data collection and analysis, decision to publish, or preparation of the manuscript.”
  d. If any authors received a salary from any of your funders, please state which authors and which funders.*

**We have updated the financial disclosure in accordance with these guidelines. In addition to updating the entry in the online submission platform, we have added this to the manuscript as a section before the abstract.**

- *4. Please provide separate figure files in .tif or .eps format.*

**We have uploaded each figure file in .tif format alongside the manuscript.**

- *5. We have noticed that you have uploaded Supporting Information files, but you have not included a list of legends. Please add a full list of legends for your Supporting Information files after the references list.*

**A list of legends has been added after references to identify the tables in the supplemental materials.**

- *6. In the online submission form, you indicated that "All statistical coding files are available upon request. The data underlying this article cannot be shared publicly due to IRB and ethical constraints. Data may be shared upon reasonable request to the corresponding and joint senior authors.".*

*All PLOS journals now require all data underlying the findings described in their manuscript to be freely available to other researchers, either 
1. In a public repository, 
2. Within the manuscript itself, or 
3. Uploaded as supplementary information.*

*This policy applies to all data except where public deposition would breach compliance with the protocol approved by your research ethics board. If your data cannot be made publicly available for ethical or legal reasons (e.g., public availability would compromise patient privacy), please explain your reasons by return email and your exemption request will be escalated to the editor for approval. Your exemption request will be handled independently and will not hold up the peer review process, but will need to be resolved should your manuscript be accepted for publication. One of the Editorial team will then be in touch if there are any issues.*

**Given the ethical protocols, we are unable to share data directly but have outlined the protocol that interested researchers must follow in order to get access. We have added this information to a Data Availability statement before the abstract (Page 2, Line 12) and have reached out directly to the editorial team to receive an exemption from this requirement.**

- *Please review your reference list to ensure that it is complete and correct. If you have cited papers that have been retracted, please include the rationale for doing so in the manuscript text, or remove these references and replace them with relevant current references. Any changes to the reference list should be mentioned in the rebuttal letter that accompanies your revised manuscript. If you need to cite a retracted article, indicate the article’s retracted status in the References list and also include a citation and full reference for the retraction notice.*

**We have reviewed the reference list to confirm it is correct. No articles which have been cited have been retracted. We have added a few new references which are largely methodological in nature to support the expanded Discussion section:**

**47.	Green DP, Lin W, Gerber C. Optimal Allocation of Interviews to Baseline and Endline Surveys in Place-Based Randomized Trials and Quasi-Experiments. Eval Rev. 2018 Aug;42(4):391–422. doi:10.1177/0193841X18799128**

**48.	Bailey DH, Duncan GJ, Cunha F, Foorman BR, Yeager DS. Persistence and Fade-Out of Educational-Intervention Effects: Mechanisms and Potential Solutions. Psychol Sci Public Interest. 2020 Oct;21(2):55–97. doi:10.1177/1529100620915848**

**49.	Angrist JD, Pischke JS. Mostly harmless econometrics: an empiricist’s companion. Princeton: Princeton University Press; 2009. 373 p.**

Review 1 comments

- *Reviewer #1: The study aimed to examine the ability of assessments of children’s early psychomotor development to predict developmental and learning outcomes at later ages. Please clarify all the abbreviations in the text and delete all personal pronouns (such as “we”, "our" and "us"). The research correctly uses the scientific method and was conducted in such a way that the results can be trusted. The paper is comprehensible.*

**Thank you for the positive comments towards this research and its comprehensibility. While we understand that, historically, academic publishing avoided using personal pronouns in writing and preferred passive voice, we prefer retaining a more active style of writing. This appears fairly common in recent Plos Global Public Health articles (e.g.** [**here**](https://journals.plos.org/globalpublichealth/article?id=10.1371/journal.pgph.0005696) **and** [**here**](https://journals.plos.org/globalpublichealth/article?id=10.1371/journal.pgph.0006417)**). If the editor feels differently, we can revisit this and revise this stylistic voice.**

- *In general, outcome measures derived from interviews and/or questionnaires administered to parents/caregivers are generally considered unreliable with respect to the child’s actual overall condition. Furthermore, the great variability and unpredictability, as well as the rapid evolution of psychomotor skill development — typical of very young children — must be taken into account when assessing using standardized tests, in order for them to be reliable.*

**We appreciate that our paper has highlighted some of the drawbacks to using assessments like CREDI. We feel like the overall message was perhaps a bit too pessimistic. We have expanded our Discussion section to clarify our position regarding these assessments, when they are useful, and when users should be careful. In general, we believe that CREDI is still a valid cross-sectional indicator of development, but that ECD at this age is an inherently malleable and variable construct, limiting the predictive power of any assessment.**

- *The study will be of interest to the readers of PLOS Global Public Health as it contributes to the growing body of evidence about the study of outcome measures in early childhood. If adequately modified with the revisions reported, it will certainly be a valid study acceptable for publication and useful for research in this field. The reviewer is available for any further clarifications.*

**Thank you again for the positive comments about our manuscript. We hope that the revisions in this submission help clarify our position regarding the utility of assessments for children under the age of three. We agree that these scores should be used cautiously and should not be used as substitutes for later developmental assessments, but believe that they are picking up on meaningful variation in children’s developmental competencies in ways that proxy measures do not and thus are still useful to study the effects of programs and policies affecting ECD.**

Review 2 comments

- *This study explored the predictive value of caregiver reported assessment of early child development and examined the concurrent validity between the IDELA and ECDI2030 assessment methods.
  Abstract- Adequate
  Methodology and statistical analysis- appropriate*

**Thank you for the positive comments on our methods and the utility of the study.**

- *There are however minor typographical errors and some unclear statements;*
  - *Page 5, line 11*

**We have revised this statement to “There has been less study on the predictive validity of ECD assessments for children under the age of three, but most researchers find a similar situation to that found in high-income countries: weaker predictive power assessments with younger children than assessments with preschool-aged children.”**

- *Page 12, line 6*

**Thank you for pointing this out. This was a link to the Supplemental Materials that broke when we moved them to a separate document. We have updated the text to: “The full results of these regression models are presented in the Supplemental Materials (Appendix S1) and estimates of** $\boldsymbol{\beta}_{\boldsymbol{1}}$**are presented below in Table 2”**

- *Page 18, lines 4 and 5*

**This was another linking error. We have updated the text to read “The full results of this model are reported in the Supplemental Materials (Appendix S2) and summarized below in Table 5”**

- *Figure captions are generally placed below the figures.*

**Table and Figure captions have been moved to below each table/figure.**

- *Generally, the manuscript is well written with clarity and coherence with the conclusions consistent with the findings presented.*

**We appreciate this positive comment!**
