# Supplementary material for: Predictive and concurrent validity of caregiver reported early childhood development assessments: A prospective cohort study of Tanzanian children
Source: PLOS Glob Public Health. 2026 Aug 12;6(8):e0006442. doi: 10.1371/journal.pgph.0006442 (PMC13466049; doi:10.1371/journal.pgph.0006442)
Supplement: Peer review history

# Peer review: Decision Letter 0

**DOI:** 10.1371/journal.pgph.0006442.r001

**Authors:** Nafis Faizi

8 Jun 2026

PGPH-D-26-01333

Predictive and concurrent validity of caregiver reported early childhood development assessments: A prospective cohort study of Tanzanian children

PLOS Global Public Health

Dear Dr. Seiden,

Thank you for submitting your manuscript to PLOS Global Public Health. After careful consideration, we feel that it has merit and good potential but does not fully meet PLOS Global Public Health’s publication criteria as it currently stands. Therefore, we invite you to submit a revised version of the manuscript that addresses the points raised during the review process.

Please ensure that the paper is thoroughly revised in terms of explaining the abbreviations and other typographical errors (as pointed by reviewers) in addition to improvement in discussion. The discussion is appropriate in terms of the explanation of the results but does not go beyond it, in terms of explaining the wider implications for ECD measures and their use in LMICs. The discussion would be strengthened with the reflection on the measures (including the ones studied) in the presence of other measure; the practical implications of the findings of the caregiver reported childhood measures and also the future directions of research (like exploring the evidence for understanding the pessimistic or optimistic point of views more clearly).

In conclusion you raise valid points on the variability of measures and how young children's performance is not deterministic but this point has not been well explained in the discussion. I think it warrants a discussion so that the readers can clearly understand what you mean by 'ongoing intervention and support'

Please submit your revised manuscript by Jul 08 2026 11:59PM. If you will need more time than this to complete your revisions, please reply to this message or contact the journal office at globalpubhealth@plos.org. When you're ready to submit your revision, log on to https://www.editorialmanager.com/pgph/ and select the 'Submissions Needing Revision' folder to locate your manuscript file.

Please include the following items when submitting your revised manuscript:

- A letter that responds to each point raised by the editor and reviewer(s). You should upload this letter as a separate file labeled 'Response to Reviewers'.
- A marked-up copy of your manuscript that highlights changes made to the original version. You should upload this as a separate file labeled 'Revised Manuscript with Track Changes'.
- An unmarked version of your revised paper without tracked changes. You should upload this as a separate file labeled 'Manuscript'.

Guidelines for resubmitting your figure files are available below the reviewer comments at the end of this letter.

As the corresponding author, your ORCID iD is verified in the submission system and will appear in the published article. PLOS supports the use of ORCID, and we encourage all coauthors to register for an ORCID iD and use it as well. Please encourage your coauthors to verify their ORCID iD within the submission system before final acceptance, as unverified ORCID iDs will not appear in the published article. *Only*  the individual author can complete the verification step; PLOS staff *cannot*  verify ORCID iDs on behalf of authors.

We look forward to receiving your revised manuscript.

Kind regards,

Nafis Faizi, MD, MPH

Academic Editor

PLOS Global Public Health

Journal Requirements:

1. Please include a complete copy of PLOS’ questionnaire on inclusivity in global research in your revised manuscript. Our policy for research in this area aims to improve transparency in the reporting of research performed outside of researchers’ own country or community. The policy applies to researchers who have travelled to a different country to conduct research, research with Indigenous populations or their lands, and research on cultural artefacts. The questionnaire can also be requested at the journal’s discretion for any other submissions, even if these conditions are not met. Please find more information on the policy and a link to download a blank copy of the questionnaire here: https://journals.plos.org/globalpublichealth/s/best-practices-in-research-reporting. Please upload a completed version of your questionnaire as Supporting Information when you resubmit your manuscript.

2. Please ensure that your Ethics Statement is available in its entirety at the beginning of your Methods section, under a subheading 'Ethics Statement'.

3. Please amend your detailed Financial Disclosure statement. This is published with the article. It must therefore be completed in full sentences and contain the exact wording you wish to be published.

a. Please clarify all sources of funding (financial or material support) for your study. List the grants (with grant number) or organizations (with url) that supported your study, including funding received from your institution.

b. State the initials, alongside each funding source, of each author to receive each grant.

c. State what role the funders took in the study. If the funders had no role in your study, please state: “The funders had no role in study design, data collection and analysis, decision to publish, or preparation of the manuscript.”

d. If any authors received a salary from any of your funders, please state which authors and which funders.

4. Please provide separate figure files in .tif or .eps format.

For more information about figure files please see our guidelines:  LINK

https://journals.plos.org/globalpublichealth/s/figures

https://journals.plos.org/globalpublichealth/s/figures#loc-file-requirements

5. We have noticed that you have uploaded Supporting Information files, but you have not included a list of legends. Please add a full list of legends for your Supporting Information files after the references list.

6. In the online submission form, you indicated that "All statistical coding files are available upon request. The data underlying this article cannot be shared publicly due to IRB and ethical constraints. Data may be shared upon reasonable request to the corresponding and joint senior authors.".

All PLOS journals now require all data underlying the findings described in their manuscript to be freely available to other researchers, either

1. In a public repository,

2. Within the manuscript itself, or

3. Uploaded as supplementary information.

This policy applies to all data except where public deposition would breach compliance with the protocol approved by your research ethics board. If your data cannot be made publicly available for ethical or legal reasons (e.g., public availability would compromise patient privacy), please explain your reasons by return email and your exemption request will be escalated to the editor for approval. Your exemption request will be handled independently and will not hold up the peer review process, but will need to be resolved should your manuscript be accepted for publication. One of the Editorial team will then be in touch if there are any issues.

If the reviewer comments include a recommendation to cite specific previously published works, please review and evaluate these publications to determine whether they are relevant and should be cited. There is no requirement to cite these works unless the editor has indicated otherwise.

Please review your reference list to ensure that it is complete and correct. If you have cited papers that have been retracted, please include the rationale for doing so in the manuscript text, or remove these references and replace them with relevant current references. Any changes to the reference list should be mentioned in the rebuttal letter that accompanies your revised manuscript. If you need to cite a retracted article, indicate the article’s retracted status in the References list and also include a citation and full reference for the retraction notice.

Additional Editor Comments (if provided):

[Note: HTML markup is below. Please do not edit.]

Reviewers' comments:

Reviewer's Responses to Questions

**Comments to the Author**

1. Does this manuscript meet PLOS Global Public Health’s publication criteria?

Reviewer #1: Yes

Reviewer #2: Yes

**********

2. Has the statistical analysis been performed appropriately and rigorously?

Reviewer #1: I don't know

Reviewer #2: Yes

**********

3. Have the authors made all data underlying the findings in their manuscript fully available (please refer to the Data Availability Statement at the start of the manuscript PDF file)?

The PLOS Data policy

Reviewer #1: Yes

Reviewer #2: No

**********

4. Is the manuscript presented in an intelligible fashion and written in standard English?

Reviewer #1: Yes

Reviewer #2: Yes

**********

Reviewer #1: The study aimed to examine the ability of assessments of children’s early psychomotor development to predict developmental and learning outcomes at later ages.

Please clarify all the abbreviations in the text and delete all personal pronouns (such as “we”, "our" and "us"). The research correctly uses the scientific method and was conducted in such a way that the results can be trusted. The paper is comprehensible.

In general, outcome measures derived from interviews and/or questionnaires administered to parents/caregivers are generally considered unreliable with respect to the child’s actual overall condition. Furthermore, the great variability and unpredictability, as well as the rapid evolution of psychomotor skill development — typical of very young children — must be taken into account when assessing using standardized tests, in order for them to be reliable.

The study will be of interest to the readers of PLOS Global Public Health as it contributes to the growing body of evidence about the study of outcome measures in early childhood. If adequately modified with the revisions reported, it will certainly be a valid study acceptable for publication and useful for research in this field.

The reviewer is available for any further clarifications.

Reviewer #2: This study explored the predictive value of caregiver reported assessment of early child development and examined the concurrent validity between the IDELA and ECDI2030 assessment methods.

Abstract- Adequate

Methodology and statistical analysis- appropriate

There are however minor typographical errors and some unclear statements;

Page 5, line 11

Page 12, line 6

Page 18, lines 4 and 5

Figure captions are generally placed below the figures.

Generally, the manuscript is well written with clarity and coherence with the conclusions consistent with the findings presented.

**********

what does this mean?). If published, this will include your full peer review and any attached files.

**Do you want your identity to be public for this peer review?**  If you choose “no”, your identity will remain anonymous but your review may still be made public.

For information about this choice, including consent withdrawal, please see our Privacy Policy

Reviewer #1: **Yes:** Rita Abrunzo

Reviewer #2: No

**********

[NOTE: If reviewer comments were submitted as an attachment file, they will be attached to this email and accessible via the submission site. Please log into your account, locate the manuscript record, and check for the action link "View Attachments". If this link does not appear, there are no attachment files.]

Attachment Submitted filename: PDF RR_PGPH-D-26-01333.pdf

# Author comment: Author response to Decision Letter 1

**DOI:** 10.1371/journal.pgph.0006442.r002

16 Jul 2026

Attachment Submitted filename: PGPH-D-26-01333_Response to Reviewers.docx

# Editor report: Decision Letter 1

**DOI:** 10.1371/journal.pgph.0006442.r003

**Authors:** Nafis Faizi

24 Jul 2026

Predictive and concurrent validity of caregiver reported early childhood development assessments: A prospective cohort study of Tanzanian children

PGPH-D-26-01333R1

Dear Seiden,

We are pleased to inform you that your manuscript 'Predictive and concurrent validity of caregiver reported early childhood development assessments: A prospective cohort study of Tanzanian children' has been provisionally accepted for publication in PLOS Global Public Health.

Before your manuscript can be formally accepted you will need to complete some formatting changes, which you will receive in a follow up email. A member of our team will be in touch with a set of requests.

Please note that your manuscript will not be scheduled for publication until you have made the required changes, so a swift response is appreciated.

IMPORTANT: The editorial review process is now complete. PLOS will only permit corrections to spelling, formatting or significant scientific errors from this point onwards. Requests for major changes, or any which affect the scientific understanding of your work, will cause delays to the publication date of your manuscript.

If your institution or institutions have a press office, please notify them about your upcoming paper to help maximize its impact. If they'll be preparing press materials, please inform our press team as soon as possible -- no later than 48 hours after receiving the formal acceptance. Your manuscript will remain under strict press embargo until 2 pm Eastern Time on the date of publication. For more information, please contact globalpubhealth@plos.org.

Thank you again for supporting Open Access publishing; we are looking forward to publishing your work in PLOS Global Public Health.

Best regards,

Nafis Faizi, MD, MPH

Academic Editor

PLOS Global Public Health

***********************************************************

Reviewer Comments (if any, and for reference):
